# “Directive Approach” for Chinese Clients Receiving Psychotherapy: Is That Really a Priority?

**DOI:** 10.3389/fpsyg.2013.00049

**Published:** 2013-02-13

**Authors:** Chi Ting Connie Ng, Susan James

**Affiliations:** ^1^Department of Educational and Counselling Psychology, and Special Education, Faculty of Education, University of British ColumbiaVancouver, BC, Canada

**Keywords:** directive approach, Chinese, psychotherapy, culture, ethnography

## Abstract

The academic literature often suggests that Chinese people prefer directive approaches in therapy. However, studies on this topic are often based on therapists’ self-reports: clients’ perceptions are rarely considered. What does “directive approach” mean? Is it what clients prefer? Using cultural psychology and medical anthropology as a theoretical framework, the ethnography explored the experience of psychotherapy from Chinese clients’ perspectives. Specifically, using ethnographic interview, eight informants, two male and six female, ranging in age from 40 to 55, were interviewed twice in-depth about their experiences of seeing Chinese therapists. All informants are Chinese immigrants who reside in a major Canadian city and saw at least one Chinese therapist in a community counseling agency within 1 year prior to the interview. In the first interview, informants created group of cards describing a list of hypothesized cultural knowledge regarding psychotherapy. After initial data analysis, the cards were presented to the informants in the second interviews, in which they confirmed and/or rejected the hypotheses by grouping, reorganizing, and ranking the cards. In the end each informant created a number of mind-maps with the cards, which served as a representation of informants’ psychological reality of psychotherapy based on their ordinary language. The maps were then further analyzed for themes among informants. Results suggest that clients appreciate therapists who “give homework,” “analyze their problems,” “talk about strategies that other clients have found useful,” “chat,” and “provide resources.” Results also highlight informants’ understanding of their own responsibility for the therapeutic relationship which has never been documented before and has important clinical implications.

## Introduction

This article outlines our study into Chinese immigrant clients’ perspectives and experiences with psychotherapy in Canadian contexts. There appears to be little information regarding Chinese clients’ perceptions of therapy in the available literature, and yet as the North American population continues to diversify, therapists and other mental health professionals have an obligation to examine cultural sensitivity issues in therapeutic practice and to develop multicultural counseling competence (Carlson et al., 1998). Over the past few decades, the importance of multicultural education has been stressed in mandates of professional associations (Speight et al., 1995), and these associations have provided guidelines to help therapists serve clients who belong to non-dominant cultures (Canadian Psychological Association, 2001; American Psychological Association, 2002). Specific to our study, Chinese people constitute one of the largest visible minority groups in Canada (Colin, 2001), and Canadian therapists are likely to be working with clients from Chinese cultural backgrounds. Hence, it is important to explore Chinese clients’ experiences and perceptions of psychotherapy. Such knowledge might help therapists improve utilization and decrease premature termination rate.

However, most suggestions on working with Chinese clients are based on professional observations and opinions. Only four studies have specifically explored effective therapeutic approaches with Chinese clients (i.e., Lin, 2001; Wei and Heppner, 2005; Cao, 2008; Kuo et al., 2011), and these studies have significant limitations. They are often based on experts’ self-reports or interpretations of data through theories and concepts in the field of psychotherapy in the west. Perceptions and interpretations through clients’ cultural lens and knowledge are rarely included – even though a therapeutic approach can be effective only when the client perceives and believes so. In addition, even if Chinese client participants’ perceptions were taken into consideration, these studies mainly rely on indirect measures of client experience developed to assess what investigators think are important about client experiences. There is also no indication that the measures were validated for their cross-cultural applicability in therapeutic relationships, and thus might not have captured the unique aspects of psychotherapy in Chinese culture.

Culture is “shared constraints that limit the behavioral repertoire available to members of a certain group in a way different from individuals belonging to some other group” (Poortinga, 1990, p. 6). Given that ecological forces are the primary shapers of cultural forms, which in turn shape behaviors, it is possible to define culture as “the totality of whatever all persons learn from all other persons” (Segall et al., 1990, p. 26). Culture is a tool that helps members in a group define their reality and worldview (Chung and Bemak, 2002) and that guides their patterns of thoughts and actions (Pedersen, 1991). Yet, culture is not a fixed entity; the boundary between one culture and another can be fluid for several reasons. Pedersen (1991) noted that behaviors can be explained both in terms of learned perspectives that are unique to a particular culture and in terms of common ground universals that are shared across cultures. In addition, people from the same ethnic group can experience cultural differences, and even a single individual may change his or her cultural reference group across time – from gender to age, to socioeconomic status, to nationality or ethnicity, or to one or another affiliation (Pedersen, 1991). Therefore, while the following discussion may tend to imply cultural differences by nationality or ethnicity, as many scholars indicted below did, and while participants in many of the studies were selected because of their ethnic backgrounds, we recognize the complex differences amongst people from or between every cultural group.

Gabrenya and Hwang (1996) noted that the Chinese are profoundly influenced by the social philosophy attributed to Confucius (
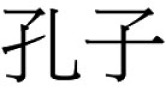
). Confucianism is a system that was developed in a time of chaos in order to allow Chinese society a modicum of harmony in the embrace of inescapable hierarchy. Confucian concepts serve as a useful framework for describing the values that guide Chinese people’s social behaviors in Chinese society. Chinese culture is relation-oriented, with social relationships and roles constituting the core of the self (Gabrenya and Hwang, 1996). The family is the basic unit of society (Goodwin and Tang, 1996): family is perceived as the “great self” (
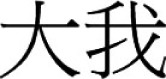
), and the individual, which is the “small self” (
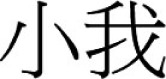
), is embedded within the family (Bedford and Hwang, 2003). In contrast to the emphasis on individual autonomy in western culture, the Chinese believe that an individual has the responsibility to preserve the functionality of the family and groups to which the individual belongs. In Chinese culture, personal identity is judged by how one behaves according to his or her relation to the group and the environment (Hsiao et al., 2006), and this behavior is often determined by external factors such as group interests or socially approved standards of excellence.

People in western individualistic culture tend to favor a low-context communication style that affirms self-identify or accomplishes individual needs and goals, level of directness, explicitness, and verbal expressiveness (Gao et al., 1996). Chinese people on the other hand, due to the socially oriented self in Chinese culture, tend to engage in a high-context communication style, which involves indirectness, implicitness, and non-verbal expression. One crucial aspect of Chinese communication is “face-saving,” which serves to preserve interpersonal harmony (Gao et al., 1996). Face refers to one’s moral integrity as a civilized person; losing face makes it impossible for the individual to function properly in the community (Gabrenya and Hwang, 1996). An indirect verbal style allows one to negotiate meaning with others in interpersonal relationships, to protect face, and to help preserve existing relationships among individuals without damaging group harmony (Gao et al., 1996).

Research investigating these cultural differences suggests that Chinese people and westerners may differ in the forms of social and emotional support they find helpful. For instance, Burleson and Mortenson (2003) found that Chinese participants valued comforting messages with a low level of person centeredness more than did Americans and perceived escape and dismiss coping behaviors more positively than did Americans. Also, in a review of studies on culture and social support, Kim et al. (2008) discovered that, compared to European Americans, Asians and Asian Americans, including Chinese people, are more reluctant to overtly solicit support from others and are more likely to rely on forms of support that avoid explicitly revealing personal stressful events and feelings of distress. This is because they may worry about the potentially negative relational consequences of such behaviors, such as disturbing group harmony, burdening others, or receiving criticism from others. Therefore, while westerners may focus on directly addressing their personal distress and issues, Chinese people tend to prefer support that maintains social harmony by strengthening personal composure and social functioning in order to avoid face loss and embarrassment (Chang, 2001).

Kleinman (1978, 2004), working from an anthropological framework, asserted that culture influences all aspects of clinical processes in psychiatry. Kleinman (1978) noted that different cultural groups often use different explanatory models, which are socially constructed clinical realities, to manage episodes of illness. People of different cultures, for example, attach different meanings and descriptions to the same syndrome, and have different views of the nature of the problem, expectations about the type and style of treatment, and doctor-patient interactions. Although Kleinman’s work is based on psychiatry, it is applicable to psychotherapist-client relationships since they are often similar to psychiatrist-patient relationships or traditional healer-patient relationships. The only difference is that the former deals with mental health issues from a psychological perspective, whereas the latter deals with the issues from a medical perspective and may include and describe medication.

Chinese people and westerners differ in the presentation of depressive symptoms (Shweder, 1991; Lee et al., 2007), and they may also differ in their expectations of client-therapist interactions and types of intervention. Specifically, Chinese clients tend to expect directive, goal oriented, time limited, and pragmatic approaches in therapy (Leong, 1986; Sue and Zane, 1987; Lin, 2001; Chong and Liu, 2002). Previous studies conducted with participants with Asian ethic background, which included Chinese participants, have supported this finding (e.g., Gim et al., 1991; Kim et al., 2002; Li and Kim, 2004).

There is a paucity of research that specifically focuses on Chinese people in a therapeutic context. Limited evidence, similar to findings that emerged from Asian participants, revealed that Chinese clients tend to perceive directive therapeutic styles as most effective. Specifically, studies showed that Chinese clients appeared to prefer a therapeutic style characterized by information and solution offering, action taking, problem solving, and an orientation toward presence (Lin, 2001; Li and Kim, 2004; Wei and Heppner, 2005; Cao, 2008; Kuo et al., 2011). For example, by combining quantitative and qualitative research methods, Wei and Heppner (2005) investigated therapist and client predictors of the initial working alliance with 31 counseling dyads from in Taiwan. They found that client participants perceived therapists to be especially credible when the therapist provided strategies on problem solving, such as encouraging clients to manage their problems, giving clients suggestions on how to cope with their issues, and providing concrete ideas on how to proceed. As well, these studies indicated that Chinese clients prefer therapists who emphasize collectivism (Kuo et al., 2011) and client confidentiality (Cao, 2008; Kuo et al., 2011).

In addition to therapeutic style, Sue and Sue (1990) suggested that, in traditional Asian cultures, therapists are typically considered authority figures, experts, or individuals who can be relied upon and trusted. Empirical evidence from studies involving Chinese participants supports this suggestion (e.g., Mau and Jepsen, 1988; Lin, 2001; Zhang et al., 2001; Kuo et al., 2011). For instance, through open-ended surveys, Zhang et al. (2001) conducted an investigation on Chinese people’s expectations for psychotherapy using Chinese visitors recruited from the clinics of local hospitals in China. The authors found that these visitors expected therapists to be profoundly knowledgeable and experienced, skillful, affable, patriarchal and experienced, talkative, friendly, similar to tutors, and to have a high moral sense. Another study showed that while American graduate students perceived therapists mostly as listeners, native Chinese graduate students in the study saw therapists as experts (Mau and Jepsen, 1988).

On the other hand, while Chinese clients might prefer a pragmatic therapeutic approach, western counseling philosophy is mainly built on Caucasian middle-class culture and tends to focus on individualism, the establishment of relationships, attaining insights, expressing emotions, and an orientation toward the future (Lin, 2001). Studies showed that people from western Euro-American cultures seem to prefer a counseling style that aligns with western counseling philosophy. Specifically, by analyzing research literature concerning clients’ notions on the helpful aspects of therapy, Elliott and James (1989) found five themes: (a) therapists were facilitative, (b) client self-expression was permitted, (c) therapist-client relationships were supportive, (d) self-understanding/insight were encouraged, and (e) therapists encouraged additional therapy practice. A recent Canadian study using a combination of qualitative and quantitative approaches (Paulson et al., 1999) and a qualitative study conducted in New Zealand (Manthei, 2007) revealed similar themes.

In sum, research suggests that Chinese clients experience a different clinical reality than clients with western cultural backgrounds and therapists trained in western counseling approaches. This disparate clinical reality includes differences in symptom expression, preferred therapeutic approaches, and expectations for therapists. For instance, Chinese clients may feel comfortable with a practical and directive therapeutic style, whereas mainstream western clients may favor an insight-oriented and non-directive style. As a result, Chinese people’s preferred therapeutic style may be at odds with the style likely favored by clients and practitioners from western cultures. Chinese counseling clients living in the west may feel lost because they have never learned how to react to therapeutic approaches common to western mainstream counseling contexts. When clients’ and therapists’ clinical realities reflect conflicting cultural backgrounds, therapeutic disengagement, patient non-compliance and dissatisfaction, and inappropriate intervention may result (Kleinman, 1978). There are relatively high under-utilization and premature termination rates among Asian American clients compared to their Caucasian counterparts (e.g., Sue et al., 1991; Jiang and Wang, 2003). One possible explanation for this is that Asian clients in western settings may feel misunderstood by their culturally different therapist and therefore lose motivation to continue therapy.

Given that there appears to be little information regarding Chinese clients’ experiences of therapy in the available literature, we questioned how Chinese clients conceptualize and articulate their therapeutic experiences according to their worldview and native language. Therefore, the purpose of our study was to understand Chinese immigrants’ perspectives and experiences with psychotherapy in a Canadian mental health context. The main research question for the present study was “How do Chinese clients experience psychotherapy?”

## Materials and Methods

### Ethnographic interview

Cultural psychologists (Shweder, 1991) and medical anthropologists (Kleinman, 2004) use qualitative methods such as ethnography when a topic or cultural group is has not been researched previously. Often when clinical psychologists start to do research with a cultural group with which they are not familiar, they explore whether their research questions make sense in that cultural context with the help of local researchers. We did this type of exploration and documented that experience by doing ethnography. The advantage of doing ethnography is that the researcher’s impressions are taken back to the participants to see if they are correct, which is a crucial step to maintain the validity of the project.

Since little is known about Chinese clients’ perceptions of therapy, a qualitative research method, namely Spradley’s (1979) ethnographic interview, was chosen for this study. Spradley’s 12-step sequence of ethnographic research consists of procedures pertinent to informant recruitment, types of interview questions, data analysis, and writing an ethnography. Specifically, this method uses the language of the informants to explore in detail their cultural context, the domains they identify as salient, and the relationships between the domains. Ethnographic interview allowed us to understand the concept of psychotherapy by exploring informants’ experiences of seeing Chinese therapists through their points of view and language.

### Recruitment criteria

An individual who satisfied the following eligibility criteria could participate in the study. The participant must: (a) currently not be in therapy, (b) have been individuals of Chinese ethnic background and who immigrated to Canada after the age of 15, (c) have been 19 years of age or older, (d) have been in therapy within 1 year prior to the interview, (e) have completed at least three sessions of therapy with the same therapist, and (f) have seen a Chinese therapist.

We recruited Chinese informants who had seen a Chinese therapist because it would enable us to obtain an original and rich picture of a clinical healing reality in Chinese culture. Therapeutic relationships between Chinese clients and Chinese therapists are likely to reflect Chinese clients’ expectations of counseling. In turn, results from this study will inform current multicultural counseling theories and practices.

### Informants

Eight informants – two male and six female – participated in this study. The informants’ ages ranged from 41 to 55. Five informants originated from Mainland China, whereas three were from Taiwan, Hong Kong, or Macau. Four informants reported having completed university education, and four reported having had a college or high-school level of education. Years of residence in Canada were between 4 and 27 years. All eight informants reported having seen at least one Chinese therapist in Canada in the past. Five informants mentioned having seen more than one therapist. Among them, two informants reported that they had seen Chinese therapists and non-Chinese therapists who spoke English with them. These therapists were master’s level, registered clinical counselors, registered social workers, or counseling practicum students, except two psychologists who had been visited by one informant at their private practice. Additionally, one informant had seen a therapist with unknown credentials in Mainland China. Four informants shared their knowledge about the counseling field or para-counseling services in their hometown.

Seven participants were satisfied with at least one of their therapists. One participant was unhappy with all the therapists she had seen. Informants’ presenting concerns included past trauma, mental health issues, family crises, parenting, and immigrant adjustment issues.

### Data collection and analysis

Recruitment took place in a major Canadian city. Recruitment flyers were posted at six counseling agencies that have Chinese-speaking therapists. Flyers were also posted at university counseling centers, libraries, and community centers. Moreover, we presented the research at Chinese mental health support groups. Recruitment continued until data saturation, which meant that analysis of the newest data provided no new information and that there was a distinct sense of redundancy in the interview text (Morse and Field, 1995).

Potential informants were screened over the phone to ensure they met the inclusion criteria. They were then provided with further details of the study. They were encouraged to ask any questions before committing to participating in the study, and were informed that they could withdraw from the study at any time. When potential informants who fitted the recruitment criteria were willing to participate in the study, an appropriate meeting time and a private meeting space were determined. Within 2 days following the initial contact, informants received a copy of informed consent through e-mail or by mail depending on the informant’s preference. Then, 2 days before the first interview, informants were contacted again by phone or e-mail to confirm their participation after reading the informed consent. None of the recruited informants withdrew from the study before the first interview.

All informants were interviewed in-person twice by the first author, except one informant who did the second interview by phone. The interviews varied between 30 and 105 min in length. Interview locations were determined based on informants’ preferences. Of the 16 interviews, 9 were conducted at informants’ homes; others were done at a counseling agency’s counseling room, at a coffee shop, or at the second author’s research lab. Informants were given the choice to speak in a language they felt comfortable using during the interview, as the first author, who conducted all the interviews, is fluent in English, Mandarin, and Cantonese. All informants were informed of their right to stop the interview at any time, and to skip or refuse to answer any questions. Ten interviews were conducted in Chinese (Cantonese or Mandarin). Two were done in English and four were done in a mixture of Chinese and English. Informants were informed that the interviews were audio-recorded for later analysis, and that their real names would not be used at any time and in any reports that resulted from their data. All interviews were transcribed and analyzed in the original language spoken in the interview. Data was not translated until the composition of study results.

At the beginning of the first interview, the first author explained to the informants the details of the informed consent form, which included the purpose of the study, the potential risks and benefits of their involvement, and informants’ ethical rights such as anonymity and the right to withdraw at any time without any penalty or harm. After the first author had answered all of informants’ questions, they were asked to sign the informed consent and to complete a demographic questionnaire. They were also offered community resources (e.g., counseling and mental health agencies) if they were interested.

During the first interview, informants were asked to talk about their past experience with receiving therapy. No specific type of therapeutic approach was introduced by the authors in the interviews. Table 1 provides a guide for the descriptive questions used in the first set of interviews and the rationale behind each question. Data analysis began after the first interview. Groups of cards that described a list of hypothesized cultural knowledge in informants’ terms were created. For example, one group of cards would include a list of terms regarding the activities a therapist did with an informant.

**Table 1:** **Guide for descriptive questions**.

Sequence	Question	Rationale for the question
Introduction	What brings you to therapy? 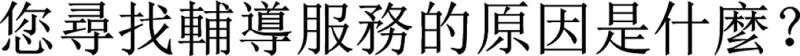	Question introduced the topic of therapy into the conversation
**GRAND TOUR QUESTION (GTQ)**		
(i) Typical grand tour question	What does a typical therapy session look like? 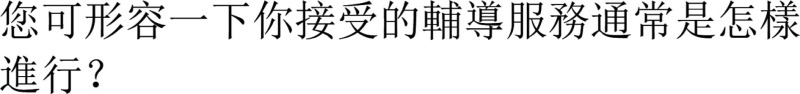	Question asks the informant to generalize, to talk about a pattern of events
	What kinds of things did you and your therapist do when you saw your therapist? 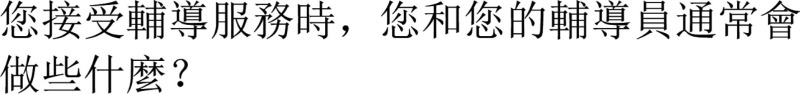	Question taps into the types of things that happened in therapy sessions
(ii) Specific Grand Tour Question	a) Could you describe a positive experience(s) or event(s) with your therapist that stood out for you the most? 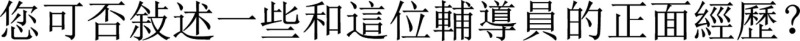	Some informants might find it difficult to describe what is typical but can easily describe experiences best known to them
	b) Could you describe a hindering experience(s) or event(s) with your therapist that stood out for you the most? 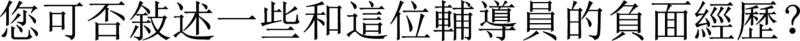	
(iii) Guided Grand Tour Questions	Imagine you are taking me into your therapy session. What are the things I would see and hear? 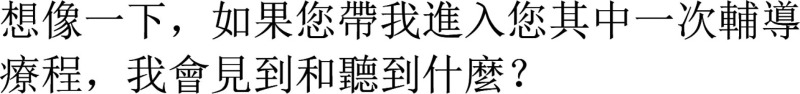	Questions that aid the informant to describe what is happening during therapy sessions
**MINI-TOUR QUESTIONS**		
	a) Can you describe 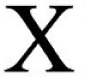 ? 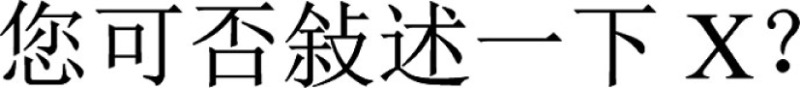	These questions usually go after the GTQ’s. They are identical to the GTQ’s except they deal with a much smaller unit of experience. The purpose of these questions is to help the informant describe specific events during therapy sessions
	b) Can you tell me more about 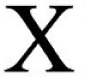 ? 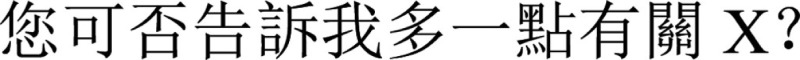	
Sample questions	Can you give me examples of 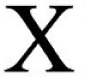 ? 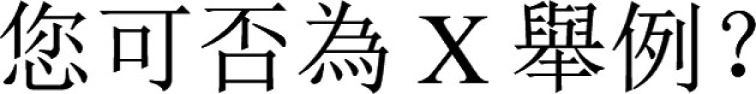	This question elicits examples of particular behaviors enacted or events that happened in the informant’s therapeutic relationship
Experience questions	If someone asks you about your experience in therapy (or in X), what would you tell him/her? 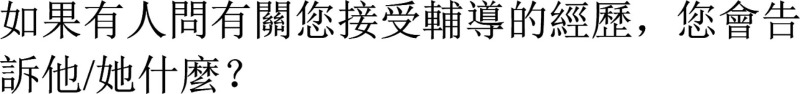	This question asks informants for any experiences they have had being a client in a therapeutic relationship. It might elicit atypical events rather than recurrent, routine ones
**NATIVE LANGUAGE QUESTIONS**		
(i) Direct language questions	How would you refer to 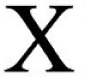 ? 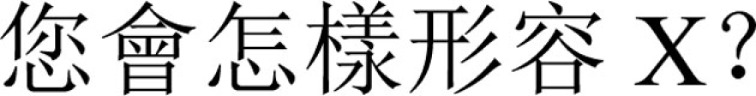	This question gets the emic language used to describe behaviors enacted or events that happened in the informant’s therapeutic relationship
(ii) Hypothetical interaction questions	Let’s say a stranger asked you about 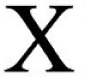 . How would you explain what 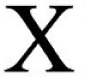 is? 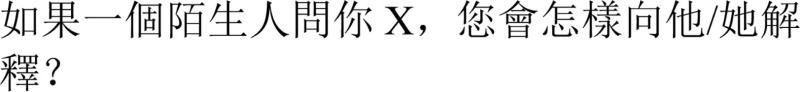	This question gets the native language used to describe behaviors enacted or events that happened in the informant’s therapeutic relationship
Other probes	Tell me more about 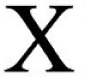 … 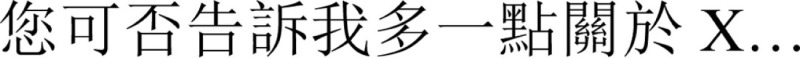	This question was used when things get quiet between the researcher and informant

**Figure 1:**
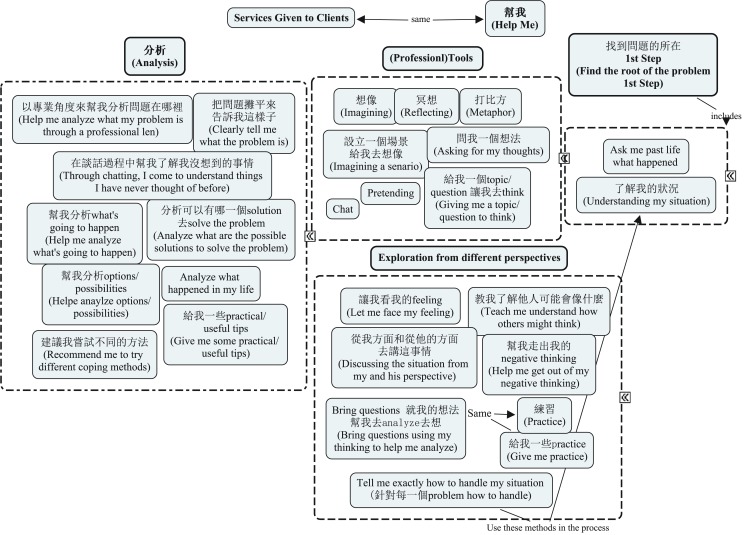
**Sample mind-map: an informant’s psychological reality of a therapist’s “services to clients.”** Bolded terms are headings of each grouping. Words in parentheses are translations of the original Chinese wordings given by the informant.

The cards were then shown to informants in the second interview, in which, through further probing, informants confirmed or rejected the hypotheses by grouping, reorganizing, and ranking the cards, thus providing new cultural knowledge. This is similar to the card sorting technique Q-sort used in quantitative research. Engaging in this activity allowed more data analysis with the informants, who helped discover and distinguish various folk terms and information about domains of knowledge in a counseling context. By the end of the second interview, informants had created several mind-maps with the cards, which served as a representation of their psychological reality of psychotherapy. (See Figure 1 for a sample mind-map, which is a duplication of an informant’s grouping of cards during the interview.) A few informants were contacted via e-mail for clarifications regarding their interview material after the second interview.

### Trustworthiness

Several credibility and trustworthiness checks were employed in the present study. First, as data analysis was in process, we attempted to identify our biases and preconceptions by referring to our journal and holding awareness of how they might influence the process of data analysis. We also discussed with each other our experiences with informants in order to stay aware of our subjectivity. Second, triangulation was achieved not only by comparing different sources of information (i.e., field notes, interview, and diary), but also by comparing different phases of the fieldwork and different points in the temporal cycles occurring in the setting (Hamersley and Atkinson, 1995). Third, referential adequacy (Eisner, 1991) was achieved through the retention of informants’ original word choices in coding and categories, and through the frequent use of quotes to illustrate categories. Fourth, respondent validation (Hamersley and Atkinson, 1995) was accomplished by having informants check the resultant categories and themes in their second interview. Finally, resultant themes were confirmed by a Chinese non-informant who is in the counseling profession and has been a counseling client with a Chinese therapist in the past.

## Results

Analyses of the interview transcriptions, informants’ mind-maps, and field notes resulted in the formation of three major themes common among the informants, most of which were termed based on informants’ word choices. When such a term was not found, a word that was linguistically close to those of informants’ was used. Names mentioned here are pseudonyms.

### Theme one: The counseling process

Many informants reported that they experienced three phases in their therapeutic experiences: (a) *understanding* (
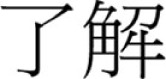
), (b) *analysis* (
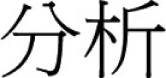
), and (c) information provision.

#### Understanding (
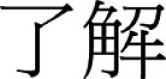
)

Six out of eight informants reported there was a phase of understanding (
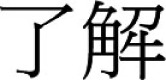
) in their therapeutic experiences. They described that their therapist would first understand their history or current situations. For example, Wong explained, “The first two sessions are for you [the therapist] to understand me and my family background. The second time you begin to talk a bit. The third time you talk a bit.”

Other informants explained the purpose of understanding a client’s background. Specifically, Zhang stated that “first thing [for a] therapist [is to know] how to figure out the problem.” And this can be achieved by *understanding my situation* (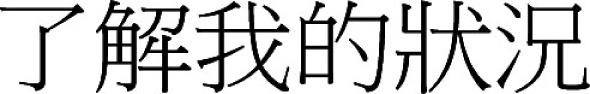), which means “figure out the big picture, the situation, and background of the client. And then identify problem.”

#### Analysis (
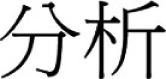
)

Another phase noted by more than half of the informants (five out of eight) is *analysis* (
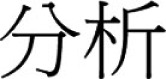
). Many informants reported that their therapist “analyzed” their situations with them by helping them understand other people’s perspectives or consider other possibilities that the informants had not considered before. In particular, Chen recounted how her therapist helped her understand the behaviors of others:

She [Chen’s therapist] helped me analyze the situation. She said that… “Okay sometimes you [the informant] don’t know what you are thinking about. Even you’re the person who is thinking, but you don’t really know.” Because sometimes we feel that we know ourselves, but in fact we don’t… That’s the origin of the problem… She wanted me to know that the first step is to know yourself very well, to analyze yourself, to analyze the problems you face, and… she also asked me a question: “I don’t know your ability of analyzing others’ behaviors, which is also important.” Because once you understand others’ purpose, you might have better strategies to deal with those negative feelings that you feel toward the behaviors.

Other informants talked about how their therapist analyzed possibilities with them. The possibilities to which the informants referred included possible causes of a situation or possible outcomes of various decisions. For instance, Lin stated:

She [Lin’s therapist] would analyze negative things. For example, if you [Lin] go with method A, what kinds of condition you might encounter. Then if you go with method B, you might have different results. She would then do some analysis. She even would go into the psychological or emotional level. She would share them with us the conditions or the responses in the cases she encountered before. I think that’s very valuable.

#### Information provision

Five out of eight informants mentioned that they appreciated their therapist providing them with information or resources related to their concerns. The information or resources appeared to be tangible or intangible material that informants could take away from their therapy sessions. For example, Wang recalled learning things from his therapist that he had been unaware of. He considered a part of therapy “is a process of getting information, getting advice, from someone who is more experienced than you.” He provided an example to illustrate this process:

I [Wang] discussed with my therapist what to do if kids make mistakes. I said, “I can’t hit. I can’t yell.” Then my therapist would use her experience, and refer to her knowledge. [She] told me what I should do. For example [she explained to me that I] can’t use physical discipline, but the level of it makes the difference. Like if you hit her, hitting her butt is okay, but slapping her face is not okay. This is a kind of advice that I didn’t know about before.

Other informants mentioned that they were given material to take away from therapy sessions. Chen stated that she appreciated how one of her therapists gave her “information after session.”

She [Chen’s therapist] gave me lots of information. I wanted to understand this. I know that it’s a theory or a strategy she learned, strategies. So I think try to give your clients some professional knowledge. It’s *very* helpful. Because that way, you try to make your client think in a professional way.

As illustrated by Lin’s comment below, other resources included information pertaining to addresses and phone numbers for various kinds of support services:

For example, I had experienced this problem in the past. So I consulted her [Lin’s therapist]… She would directly tell me. When she encounters this kind of problem, she would print out some information, and would tell me, “If you encounter this kind of problem, the Internet site is here and the address is here. What kind of agency do you need? The phone number is here and support numbers too.” She would give me a list for different problems.

Lee remarked that therapists can provide clients with options because sometimes clients “did not have the knowledge.” He further elaborated that options, for example, can include information found in library, on the Internet, or in other businesses.

Some informants mentioned that their therapists shared with them cases they had worked with in the past as references. For instance, Lin stated that she appreciated her therapist sharing outcomes of some cases with which the therapist had worked before so that she could learn from it and manage her own situation more effectively.

It appears that informants found the information acquired through therapy helpful. Without receiving information, informants’ perceived effectiveness of therapy appears to diminish. For example, Wu reported that her therapist only talked but did nothing else with her in sessions. She thought there should be some “things that she could do,” such as “homework” for her to complete, other than alleviating stress by only talking with her therapist. When asked what therapy would look like for her if she had the “homework” or “things to do,” Wu was not able provide a concrete example – she stated that she was not sure what she would be doing in sessions. However, she mentioned that going to mental health workshops (information sessions) was more helpful than going to therapy.

While it appeared that many informants perceived that therapy involves *understanding*, *analyzing*, and information provision, informants regarded the occurrences of these three phases differently. Specifically, half of the informants reported that these phases occurred in sequence in the therapeutic process. Among these informants, two reported that their therapeutic process involved their therapist first understanding them and then analyzing their situations. The other two informants stated that their therapeutic process involved their therapist analyzing their situations with them, and then “providing options” or offering them “solutions.” The other half of the informants stated that therapists should incorporate some of the phases in sessions; however, they did not mention that these phases occur in a sequential manner. Two of the four informants noted that *understanding* should be the first step in therapy, but they did not indicate if other phases should follow. The other two informants noted that two of the phases, namely *understanding* and information provision, should occur in therapy, but did not talk about them in any kind of specific order. In sum, some informants saw these phases happening sequentially and others did not.

### Theme Two: Chatting (
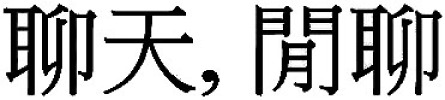
)

When asked what they usually did with their therapist, all informants reported talking, chatting, or causal chats (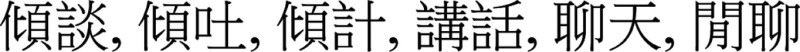) as the main mode of activity. However, informants mentioned that two components were generally incorporated into the process of chatting in therapy: (a) guidance (
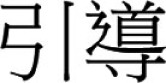
) and (b) activities.

#### Guidance (
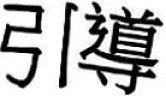
)

Although the informants stated that they and their therapist simply talked most of the time, some of them described the talks as guided conversations. Several informants praised their therapists for *smartly* (
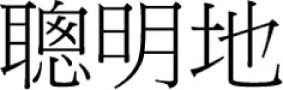
) *guiding* (
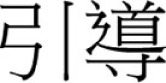
) them in conversations. For instance, Wang commented that the kind of guided talks he had with his therapist, as shown below, was *an art of conversation* (
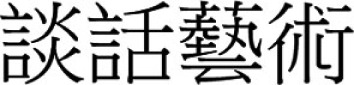
):

Initially it looks like it’s a causal talk. In fact, I can tell it’s been carefully designed. The final topic is always about parenting kids. But parenting does not mean, “You shouldn’t do this and that.” It’s through the process of talking or guiding, you realize certain things and learn the differences among them. Mostly it’s guidance.

While these informants appreciated their therapist guiding the therapeutic process, Chen talked about what happened when she felt that one of her therapists was not taking the lead:

She’s [the therapist’s] still the dominant person who is in control the situation, but not bossy. But with the Canadian therapist, I feel that she let me control the session. Because [my] talk [took] a large part of the session. She became a listener. And I did 10 sessions we yet didn’t achieve any practical goal.

Zhang succinctly echoed the comment above as she noted that she was “a follower” who “took orders” (
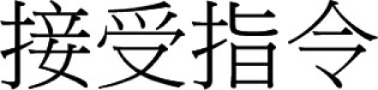
) from her therapist. On the other hand, she contended that although she may not be as knowledgeable as her therapist, her relationship with her therapist was egalitarian.

#### Activities

Besides chatting, some informants mentioned that their therapist included other exercises in sessions, such as using toys, visualizing, imagining, drawing, pretending, and practicing breathing exercises. For example, Zhang described an imagination exercise she did with her therapist:

I remember she [Zhang’s therapist] set up a situation for me to imagine and pretend that it’s a play. Then she asked for my thinking, imagining if I was that person–-what I would do if I was by myself. In the next session, she would ask for my thinking again, bringing questions to me and asking me to analyze and think accordingly, from my perspective [and] from her perspective. Then next time [she] still asked me to think and practice again. Then my thinking would change.

In fact, informants who stated that their therapy sessions were “only chatting” reported that at least some phases of the therapeutic process noted in the previous theme, such as *analyzing* and *getting information*, were incorporated in their therapy sessions.

In sum, even though many informants reported that chatting was the main mode of activity in therapy, the “chatting” appears to entail a complex level of verbal interaction between the therapist and the client. It appears that informants are likely to find therapy helpful when therapists take the lead in the chats, and when some activities, or elements of the therapeutic process noted in the previous theme, are incorporated into the chats.

### Theme three: Roles of clients and therapists

During the interviews, some informants brought up their role and their therapist’s role in therapy. We did not ask the informants questions about these two subject areas; the focus of the interviews was primarily on informants’ experiences in therapy.

When talking about clients’ role in therapy, informants mentioned two attitudes clients should possess. The first commonly mentioned attitude is the *desire* (
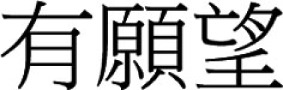
) to go to therapy. Clients need to make the conscious decision to participate in therapy and the decision needs to be voluntary. As Wang explained:

I now realize that only depending on myself is not enough. Still have to receive help from others, even though it’s a bit of an extreme way of getting help. But I choose and am willing to come [to therapy]. You have to have the desire and will. It’s like when you got sick, usually you hate taking [Chinese] medicine because it’s bitter. But you know you are sick. Even though you don’t want to drink [the medicine] you still drink it.

The second frequently mentioned attitude was *honesty* (
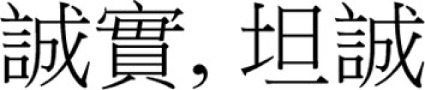
), or open-up (
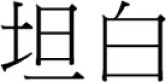
). An informant remarked that it is important that clients are honest with their therapist. If clients are not honest, therapists will not be able to appropriately provide suggestions to the clients. For instance, Wang stated:

[Clients] should not provide false information because people [therapists] might struggle. Say you go see a doctor. You know you have a headache but you said you have back pain. Then doctor would give you medicine for back pain, but you would not get well form your headache. You have to be honest.

As Zhang further expounded, some clients might not want to be honest with their therapist because they are afraid of being judged, losing face and dignity, and facing their problems:

Sometimes I would think whether I should tell him [the therapist] this, because I’m a human being and therefore selfish! I don’t want to talk about [my problems]. But if you aren’t honest, the therapist would go around with you. I feel that the difficulty is, in Chinese culture, we’re not used to honestly facing our problem. We also don’t know how… the so-called saving face/dignity.

In addition to client attitudes, some informants talked about the client-therapist relationship. When asked what makes a good therapist, some informants’ responses did not focus on the characteristics of the therapist, but on the therapist-client match instead. They noted that whether therapy works *depends on the person* (
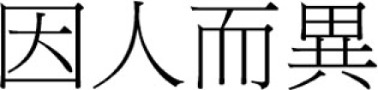
). For example, Wong noted that not everyone would find the same therapist helpful:

Everyone is different. To me may be she [the therapist] is good, but she might not be so good for another client. It’s very hard to say… Like some people like having dim-sum; some people like drinking coffee; some people like eating Western food.

Some informants stated, similarly, that therapy is *both-ways* (
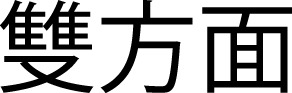
), which means both therapists and clients are contributors to the therapeutic process. Lin explained that therapy requires both the therapist and the client to participate in the conversation:

Therapy is two-way. If it’s one-way then [you would] rather find a friend, or talk to yourself. But I would say you would not see [any] effect. You would not get what you want–-having ways to solve the problem. Two-way means, within an hour conversation, you talk, she [the therapist] answers. She talks and you are willing to share more. Not one-way: only one person talking, only depending on one side, forcing you to talk.

Moreover, Lee stated that therapy has no guarantee because, in order for therapy to work, the therapist and the client need to be compatible:

Therapy is not just one-sided. I always say two-sided. The wheels have to click together. If the wheels can’t click with each other, it might become worse. Of course, the therapist is supposed to be more flexible, meaning [the therapist] should know how to click with other people. But that doesn’t mean that it always clicks.

Lee then gave an example of client-therapist match:

You [a therapist] have to consider your client. For example, you have a client who is a problematic teen. If you present yourself like a teacher, no one will pay attention to you. They won’t give a damn about you. But maybe the therapist was also a gangster in the past. He would use the attitude that he had in the past to connect with the teen. Or when he talks to the teen he wouldn’t [say], “Hey have you eaten yet? Why are you smoking?” He would never say, “Hey don’t smoke!” I [the therapist] would smoke with them. Therapists have to consider [the background of each] client.

Based on the interviews, it appears that some informants believe that the therapist is not the sole factor that makes therapy work, but rather, that successful therapy depends on the relationship between the client and the therapist.

## Discussion

Since there is little information in the literature with respect to Chinese clients’ perceptions of psychotherapy, the purpose of the present study was to explore how Chinese clients perceive and describe therapy. The resultant themes will be discussed first, and the research question noted previously will be addressed in this section.

### The counseling process and chatting

Many informants in this study reported that they experienced three phases in their therapeutic experience: (a) *understanding* (
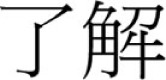
), (b) *analysis* (
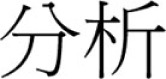
), and (c) information provision. Given that a number of informants saw these phases occurring in a sequence, it seems that some informants were describing a cultural script for therapy. In addition, it appears that these phases are present and goal oriented. This finding matches other scholars’ assertions regarding Chinese people’s preferences for a pragmatic therapeutic style (e.g., Sue and Zane, 1987; Chong and Liu, 2002; Chang et al., 2005). Chong and Liu (2002) noted a Chinese proverb that says, “The purpose of learning is for use,” which illustrates that Chinese people value practice over conceptual thinking. Thus, Chinese people may desire to gain immediate benefits or resolution to their problems from therapy and may in turn look for direct guidance from professionals to solve their problems in a short time.

The preference for pragmatic elements in therapy found in our study is also evident in the research (e.g., Wei and Heppner, 2005; Cao, 2008; Kuo et al., 2011). For instance, Cao (2008) found that Chinese college students were likely to be willing to stay in therapy and provide high ratings of therapist credibility when they perceived that therapy was directive and involved concrete and tangible techniques such as conveying information, probing for information, giving advice, directing behavior, and making interpretations. However, the comparability among these studies and ours might be limited because of the characteristics of research participants: the cited research mainly involved Chinese college students as research participants, whereas the informants in our study were mostly middle-aged members of urban Canadian communities.

The pragmatic characteristic of therapeutic process is also apparent in how informants in our study talked about what they did in therapy. Specifically, although most informants reported chatting as the main mode of activity in therapy, many noted that they also engaged in other activities. Moreover, several informants mentioned that the chatting was a guided conversation, in which the therapist took the lead and gave them information or practical resources. Hence, it appears that the chatting reported by many informants entailed a level of complexity. Such findings fit the scholarly claim (e.g., Chong and Liu, 2002) that Chinese people are likely to expect a “doing” and a practical approach in therapy, in which the therapist is perceived as an expert and a leader.

It is worthwhile to underscore the phase of *analysis* found in the current study. A number of informants reported that they appreciated their therapist helping them analyze their situations. This appreciation of the “analysis” element in therapy may be a result of the influence of Confucianism, in which an individual is characterized as a relational self in relationships and in social contexts (Chen et al., 2006): as described in the Section “Introduction,” people are part of a family and larger social systems (Nutt, 2007). Given this cultural tradition, it is not surprising that a few informants in the present study mentioned that they appreciated their therapists’ help in analyzing their situations by getting them to consider others’ perspectives. Because the informants are likely to perceive themselves as interdependent individuals, they may have thought about their concerns in relation to others, in turn preserving social harmony. They found it helpful when their therapist helped them understand others’ perspectives on their presenting issues, perhaps because this fits with their relation-oriented mindset.

### Roles of clients and therapists

Some informants in this study brought up their role as a client and the role of the therapist-client match. Some informants perceived that, in order for therapy to be effective, clients need to participate and take responsibility in the therapeutic process. Specifically, they stated that clients need to have the *desire* (
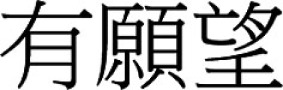
) to go to therapy and to be *honest* (
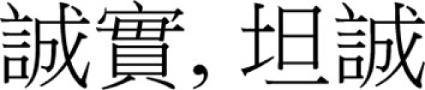
) with their therapist.

Such understandings do not seem to be reflected consistently in the scarce research. In particular, participants in Lin’s (2001) study did not seem to share the same understanding of clients’ role in therapy as the informants in the current study. Lin found that participants in her study felt that therapists are responsible for therapeutic outcomes; they did not seem to be aware of the shared responsibility between therapists and clients. On the other hand, Hou et al. (2009) found that Chinese university participants, similar to some informants in the current study, had significantly higher expectations than high-school participants in terms of client motivation, openness, and responsibility. However, the studies by Lin (2001) and Hou et al. (2009) were conducted with students, whereas informants in our study were middle-aged. Age might be a mediating factor that facilitates individuals’ understandings of clients’ role in therapy. Different research methods (i.e., empirical measures in Hou et al.’s study versus open-ended questions in Lin’s study) might also play a role in the disparate results found in these studies. Therefore, comparisons need to be made with caution.

There appears to be a discrepancy between the available existing research and this study regarding what constitutes a client’s participation in therapy. Specifically, the concept of honesty (
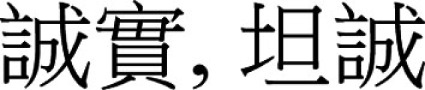
) we found is not reflected in available studies that involved Chinese clients or the mainstream Caucasian population. A similar concept, self-disclosure, is found in studies that involved participants from mainstream population (e.g., Elliott and James, 1989; Paulson et al., 1999). Although both self-disclosure and honesty involve revealing information to others, the latter has a moral connotation to it. Therefore, while informants’ perception of their role in therapy is somewhat reflected in existing research, given the very limited available studies with Chinese populations and their mixed results, it is difficult to conclude whether Chinese people are aware of their role in therapy.

The socially oriented self and high-context communication style in Chinese culture noted in the introduction may explain why some informants in our study mentioned the importance of client honesty in therapist relationships. As noted in the Section “Introduction,” Chinese people tend to engage in an indirect mode of communication in order to protect face and preserve interpersonal relationships (Gao et al., 1996). They are more likely to rely on support that does not involve explicitly disclosing personal stressful events and feelings of distress because their worries of the potentially negative relational consequences (Kim et al., 2008). Hence, they may find authentically revealing one’s self a foreign and uncomfortable task. Future research is needed to explore the relationship between Chinese people’s communication style and client honesty in therapeutic relationships.

As noted previously, Chinese cultural norms tend to foster concerns about face-saving and preference for implicit support. However, the way informants responded to their therapeutic experience seems to deviate from this cultural norm. Particularly, unlike typical supportive relationships, informants seemed to forgo their concerns about face-saving and be honest and direct with their therapist instead. In Chinese societies, members of small social groups are clearly differentiated from outsiders: privacy is often defined by the family boundary which differentiates family members from outsiders (Chan, 2000). A person’s privacy boundary has a relatively greater permeability in Chinese families than in western ones. Given this cultural tradition, one may speculate that the therapeutic relationships described by informants may be similar to familial or close relationships in Chinese culture. Informants might felt that their therapist was like an insider, a close friend, or a family member, and thus honesty or disclosing personal information became a norm in the therapeutic relationship.

The role of the therapist-client match mentioned by a few informants in the current study is generally supported by Manthei’s (2007) results. In particular, client participants in Manthei’s study perceived that they and their therapist were compatible because their perceived needs were met by their therapist or they were similar to their therapist in notable ways. Such compatibility appeared to facilitate the therapeutic process. Nevertheless, no available study was found that explores the idea of therapist-client match specifically from Chinese clients’ perspectives. Many of the available studies regarding therapist-client match involved Chinese participants as part of the study sample and were outcome research in which various therapeutic outcomes were investigated by matching different client and therapist variables. Therapist-client match, for example, included a match between therapists’ and clients’ worldviews (e.g., Kim et al., 2005), a match between therapists’ counseling styles and clients’ cultural values (e.g., Kim et al., 2002; Li and Kim, 2004), and a match between therapists’ ethnicity and clients’ cultural values (e.g., Kim and Atkinson, 2002). Therapeutic outcomes consisted of client-therapist working alliance, therapist credibility, empathy, and cross-cultural competence (e.g., Li and Kim, 2004; Kim et al., 2005, 2009). Due to the lack of information in the literature, little is known about the phenomenon of therapist-client match from Chinese clients’ perspectives. It would be interesting to know what the perceived components and the associated concepts of this phenomenon are.

In conclusion, the topics regarding the roles of clients and therapists appear to be relatively new topics in the psychotherapy literature involving the Chinese population, and thus future research is needed.

### Chinese counseling clients’ preferred therapeutic approach

It appears that many informants appreciated therapists’ attempts to understand and analyze their situations through chatting, coupled with other types of activities. After learning about the informants’ problem, the therapist would offer them resources, new perspectives, or information. Also, while informants understand their own responsibilities in therapy, they also expect the therapist to take the lead and guide the session.

Many informants described their therapeutic experience in a pragmatic manner and their descriptions support the current literature that Chinese people tend to find a directive approach of therapy effective. Study results reflect the observation that Chinese people, tend to favor a therapist who is active, directive, and present-oriented and that they may not appreciate a therapeutic style that is based on western counseling philosophy, which tends to be non-directive and insight-oriented.

The current study revealed what the “directive approach” entails based on informants’ native language and clinical reality. However, informants did not explicitly use the term “directive approach” in their discourse, although this term is well documented in the literature. As no research has explored how Chinese counseling clients would have termed the aforementioned descriptions of therapy in their native language, further research is needed. Knowledge gained from such research may help enhance communication between therapists and Chinese counseling clients, and clients’ understanding of therapy, in turn facilitating positive therapeutic outcomes.

### Implications for practice

The study results suggest several implications for practice. First, given the therapeutic process described by informants, therapists may want to stay pragmatic with Chinese clients to establish credibility. For example, therapists may not only acknowledge clients’ emotional experience, but may also balance the therapeutic process with some activities, psychoeducation, and other resources, such as a new perspective, knowledge, take-home practice, or a list of resources. Therapists can also share other clients’ experiences with their clients while maintaining client confidentiality. When Chinese clients have something concrete or cognitively stimulating, they might feel that they have accomplished something, which may motivate them to continue therapy.

Another implication that can be drawn from these results is that therapists can emphasize the role of client responsibility and the client-therapist match when working with Chinese clients. For instance, as study informants mentioned, sometimes clients might not be honest about their concerns, and therapists can educate clients about the need for honesty in order to achieve positive therapeutic outcomes. At the same time, therapists may encourage honesty by normalizing clients’ concerns and by being non-judgmental, which allows the client to save face and to feel that the therapist is an insider. Moreover, therapists can explain to clients the role of client-therapist compatibility in effective therapy.

Lastly, many informants reported that their therapist “analyzed” their situations by helping them understand other people’s perspectives. This seems to reflect Chinese people’s relational orientation, whereby group interests are prioritized over self-interest. Hence, therapists may conceptualize Chinese clients as relational individuals. Even if a Chinese client comes for individual therapy, therapists may, aside from a focus on the client *only*, also address the people involved in the client’s situation. As well, therapists can empathize with clients’ relational self; rather than only addressing clients’ feelings about themselves, therapists may also tap into how clients might feel about the relationships between themselves and other involved parties. Meanwhile, therapists can help clients understand others’ points of view. By conceptualizing Chinese clients as interdependent individuals, therapists may effectively engage them in therapy.

However, one must treat the suggestions above with caution. As discussed in the introduction, culture is a complex, fluid, and time-specific phenomenon. Thus, therapists should not presume that these suggestions are applicable to all Chinese counseling clients. One must consider other factors, such as age, gender, history, education, occupation, socioeconomic status, acculturation rate, and geographic location, which may all play a role in how a client responds to different kinds of therapist styles and therapeutic interventions.

Therefore, while therapists can develop culturally appropriate strategies for working with clients from different ethnic backgrounds, they should take clients’ individual differences and needs into consideration. One-way therapists can achieve this is to conceptualize, as delineated by Pedersen (1991), culture as a perspective in which two individuals can disagree without one being right and the other being wrong since their arguments are based on different cultural assumptions. Also, two culturally different persons’ behaviors can be very different even though they share the same expectations and goals. Conversely, the same culturally learned behaviors may have various implications for different people and even for the same individual across times and situations. To avoid misinterpretation, therapists can identify the common ground between them and their clients during sessions. Otherwise, the therapist is not likely to accurately assess the client’s concerns. As Pedersen (1991) stressed, if our understanding of cultures is narrowly defined, the relevance of culture and multicultural counseling is not likely to be appreciated.

### Limitations

There are several limitations of this study. First, study results cannot be generalized in the same way that large-scale statistical studies with fully randomized sampling may be. Specifically, study results cannot be generalized to all Chinese immigrants and other types of Asian clients and to all kinds of therapy in North America. Second, while some informants described their unsatisfying therapeutic experiences, most of them reported having positive experiences and are middle-aged. Thus, study results may be skewed and could primarily reflect therapeutic experience of some Canadian Chinese immigrants, who are likely to be motivated to change and cope with their concerns and to favor and be open to therapy. Third, while efforts have been made to ensure validity, qualitative research is inherently biased and to some degree subjective because of the researchers’ influences over the process of induction. Finally, the present study is formulated based on multicultural counseling theories and research mostly originating from western culture and thus may not be valid in psychotherapy and other forms of client-healer relationships in other Chinese societies.

### Future research

As there is limited research done concerning Chinese people’s perceptions of psychotherapy, this research can serve as a preliminary study that provides direction for future research. Specifically, a number of future research areas can be considered. First, aside from interviewing informants, researchers can add behavioral observations of therapy sessions in order to enhance the study’s consistency and identify interaction patterns between therapists and clients that might embody Chinese clients’ perceptions of therapy.

Even though current results show that informants tended to perceive therapy as effective when therapists delivered the service in a “directive” manner, the components (i.e., the counseling process and chatting) that constitute this “directive” approach are not documented in the literature. Also, informants did not explicitly use the term “directive approach” in their discourse although this term is well noted in the psychotherapy literature. Further studies are needed to verify results from the current study and to investigate what Chinese clients would have called the “directive” therapeutic approach, a term that is often used in psychotherapy in the west.

Finally, there appears to be no research that has examined one of the themes found in this study, namely Chinese clients’ perceptions on the role of the client and that of the therapist in therapy. Due to the paucity of information in the literature, understanding of this theme is limited; thus, future research is needed.

In sum, future research would help gain further understanding of how Chinese counseling clients may understand the concept of psychotherapy in the west. Such knowledge, as discussed above, may help therapists effectively communicate with Chinese clients, in turn reducing clients’ misconceptions of psychotherapy and facilitating their willingness to utilize therapy when needed.

## Conflict of Interest Statement

The authors declare that the research was conducted in the absence of any commercial or financial relationships that could be construed as a potential conflict of interest.

## References

[B1] American Psychological Association. (2002). Ethical principles of psychologists and code of conduct. Am. Psychol. 57, 1060–1073 10.1037/0003-066X.57.12.1060 12613157

[B2] Bedford O. Hwang K. K. (2003). Guilt and shame in Chinese culture: a cross-cultural framework from the perspective of morality and identity. J. Theory Soc. Behav. 33, 125–142 10.1111/1468-5914.00210

[B3] Burleson B. R. Mortenson S. R. (2003). Explaining cultural differences in evaluations of emotional support behaviors exploring the mediating influences of value systems and interaction goals. Communic. Res. 30, 113–146 10.1177/0093650202250873

[B4] Canadian Psychological Association. (2001). Guidelines for Non-Discriminatory Practice. Ottawa, ON: Author

[B5] Cao J. (2008). The Credibility of Psychotherapy: Psychological Reactance and Chinese Students’ Impressions of Directive and Nondirective Approaches. Doctoral dissertation. Retrieved from ProQuest Digital Dissertations. (New York: Publication No. AAT 3327483).

[B6] Carlson M. H. Brack C. J. Laygo R. Cohen R. Kirkscey M. (1998). An exploratory study of multicultural competence of counselors in training: support for experiential skills building. Clin. Superv. 17, 75–87 10.1300/J001v17n02_04

[B7] Chan Y.-K. (2000). Privacy in the family: its hierarchical and asymmetric nature. J. Comp. Fam. Stud. 31, 1–17

[B8] Chang D. F. Tong H. Shi Q. Zeng Q. (2005). Letting a hundred flowers bloom: counseling and psychotherapy in the People’s Republic of China. J. Ment. Health Couns. 27, 104–116

[B9] Chang E. C. (2001). “A look at the coping strategies and styles of Asian Americans: similar and different?” in Coping with Stress: Effective People and Processes, ed Snyder C. R. (New York: Oxford University Press), 222–239

[B10] Chen S. Boucher H. C. Tapias M. P. (2006). The relational self revealed: integrative conceptualization and implications for interpersonal life. Psychol. Bull. 132, 151–179 10.1037/0033-2909.132.2.151 16536640

[B11] Chong F. H.-H. Liu H.-Y. (2002). Indigenous counseling in the Chinese cultural context: experience transformed model. Asian J. Couns. 9, 49–68

[B12] Chung R. C.-Y. Bemak F. (2002). The relationship of culture and empathy in cross-cultural counselling. J. Couns. Dev. 80, 154–159 10.1002/j.1556-6678.2002.tb00173.x

[B13] Colin L. (2001). Profiles of Ethnic Communities in Canada: The Chinese Community in Canada, 2001 (No. 89-621-XIE). Ottawa, ON: Statistics Canada Available at: http://www.statcan.gc.ca/pub/89-621-x/89-621-x2006001-eng.pdf

[B14] Eisner E. W. (1991). The Enlightened Eye. New York: MacMillan

[B15] Elliott R. James E. (1989). Varieties of client experience in psychotherapy: an analysis of the literature. Clin. Psychol. Rev. 9, 443–467 10.1016/0272-7358(89)90003-2

[B16] Gabrenya W. K. Hwang K.-K. (1996). “Chinese social interaction: harmony and hierarchy on the good earth,” in The Handbook of Chinese Psychology, ed. Bond M. H. (New York: Oxford University Press), 309–321

[B17] Gao G. Ting-Toomey S. Gudykunst W. B. (1996). “Chinese communication processes,” in The Handbook of Chinese psychology, ed. Bond M. H. (New York: Oxford University Press), 280–293

[B18] Gim R. H. Atkinson D. R. Kim S. J. (1991). Asian-American acculturation, counselor ethnicity and cultural sensitivity, and ratings of counselors. J. Couns. Psychol. 38, 57–62 10.1037/0022-0167.38.1.57

[B19] Goodwin R. Tang S.-K. (1996). “Chinese personal relationships,” in The Handbook of Chinese Psychology, ed. Bond M. H. (New York: Oxford University Press), 294–308

[B20] Hamersley M. Atkinson P. (1995). Ethnography: Principles in Practice, 2nd Edn. New York: Routledge

[B21] Hou Z.-J. Zhou S.-L. Ma C. (2009). Preliminary research on high school and university students’ expectation about counseling. Chin. J. Clin. Psychol. 17, 515–517

[B22] Hsiao F. H. Klimidis S. Minas H. M. Tan E. S. (2006). Cultural attribution of mental health suffering in Chinese societies: the views of Chinese patients with mental illness and their caregivers. J. Clin. Nurs. 15, 998–1006 10.1111/j.1365-2702.2006.01331.x 16879544

[B23] Jiang G. Wang M. (2003). A study on help-seeking propensity of Chinese undergraduates [in Chinese]. Chin. J. Clin. Psychol. 11, 180–184

[B24] Kim B. S. K. Atkinson D. R. (2002). Asian American client adherence to Asian cultural values, counselor expression of cultural values, counselor ethnicity, and career counseling process. J. Couns. Psychol. 49, 3–13 10.1037/0022-0167.49.1.3

[B25] Kim B. S. K. Li L. C. Liang C. T. H. (2002). Effects of Asian American client adherence to Asian cultural values, session goal, and counselor emphasis of client expression on career counseling process. J. Couns. Psychol. 49, 342–354 10.1037/0022-0167.49.1.3

[B26] Kim B. S. K. Ng G. F. Ahn A. J. (2005). Effects of client expectation for counseling success, client-counselor worldview match, and client adherence to Asian and European American cultural values on counseling process with Asian Americans. J. Couns. Psychol. 52, 67–76 10.1037/0022-0167.52.1.67

[B27] Kim B. S. K. Ng G. F. Ahn A. J. (2009). Client adherence to Asian cultural values, common factors in counseling, and session outcome with Asian American clients at a university counseling center. J. Couns. Dev. 87, 131–142 10.1002/j.1556-6678.2009.tb00560.x

[B28] Kim H. S. Sherman D. K. Taylor S. E. (2008). Culture and social support. Am. Psychol. 63, 518–526 10.1037/0003-066X 18793039

[B29] Kleinman A. (1978). Clinical relevance of anthropological and cross-cultural research: concepts and strategies. Am. J. Psychiatry 135, 427–431 637136 10.1176/ajp.135.4.427

[B30] Kleinman A. (2004). Culture and depression. N. Engl. J. Med. 351, 951–953 10.1056/NEJMp048078 15342799

[B31] Kuo B. C. H. Hsu W.-S. Lai N.-H. (2011). Indigenous crisis counseling in Taiwan: an exploratory qualitative case study of an expert therapist. Int. J. Adv. Couns. 33, 1–21 10.1007/s10447-010-9108-y

[B32] Lee D. T. Kleinman J. Kleinman A. (2007). Rethinking depression: an ethnographic study of the experiences of depression among Chinese. Harv. Rev. Psychiatry 15, 1–8 10.1080/10673220601183915 17364968

[B33] Leong F. T. (1986). Counselling and psychotherapy with Asian-Americans: review of the literature. J. Couns. Psychol. 33, 196–206 10.1037/0022-0167.33.2.196

[B34] Li L. C. Kim B. S. K. (2004). Effects of counseling style and client adherence to Asian cultural values on counseling process with Asian American college students. J. Couns. Psychol. 51, 158–167 10.1037/0022-0167.51.2.158

[B35] Lin Y.-N. (2001). Taiwanese female university students’ perceptions of counselor effectiveness. Int. J. Adv. Couns. 23, 51–72 10.1023/A:1010671731218

[B36] Manthei R. J. (2007). Clients talk about their experience of the process of counselling. Couns. Psychol. Q. 20, 1–26 10.1080/09515070701208359

[B37] Mau W.-C. Jepsen D. A. (1988). Attitudes toward counselors and counseling processes: a comparison of Chinese and American graduate students. J. Couns. Dev. 67, 189–192 10.1002/j.1556-6676.1988.tb02090.x

[B38] Morse J. M. Field P. A. (1995). Qualitative Research Methods for Health Professionals. Thousand Oaks, CA: Sage

[B39] Nutt R. L. (2007). Implications of globalization for training in counseling psychology: presidential address. Couns. Psychol. 35, 157–171 10.1177/0011000006294671

[B40] Paulson B. L. Truscott D. Stuart J. (1999). Clients’ perceptions of helpful experiences in counseling. J. Couns. Psychol. 46, 317–324 10.1037/0022-0167.46.3.317

[B41] Pedersen P. (1991). Multiculturalism as a generic approach to counselling. J. Couns. Dev. 70, 6–12 10.1002/j.1556-6676.1991.tb01554.x

[B42] Poortinga Y. H. (1990). Towards a conceptualization of culture for psychology. Cross Cult. Psychol. Bull. 24, 2–10

[B43] Segall M. H. Dasan P. R. Berry J. W. Poortinga Y. H. (1990). Human Behavior in Global Perspective: An Introduction to Cross-Cultural Psychology. New York: Pergamon

[B44] Shweder R. A. (1991). Thinking Through Cultures: Expeditions in Cultural Psychology. Cambridge, MA: Harvard University Press

[B45] Speight S. L. Thomas A. J. Kennel R. G. Anderson M. E. (1995). Operationalizing multicultural training in doctoral programs and internships. Prof. Psychol. Res. Pr. 26, 401–406 10.1037/0735-7028.26.4.401

[B46] Spradley J. P. (1979). The Ethnographic Interview. New York: Holt, Rinehart and Winston

[B47] Sue D. W. Sue D. (1990). Counseling the Culturally Different: Theory and Practice, 1st Edn. New York: Wiley

[B48] Sue S. Fujino D. Hu L.-T. Takeuchi D. T. Zane N. W. S. (1991). Community mental health services for ethnic minority groups: a test of the cultural responsive hypothesis. J. Consult. Clin. Psychol. 59, 533–540 10.1037/0022-006X.59.4.533 1918557

[B49] Sue S. Zane N. (1987). The role of culture and cultural techniques in psychotherapy: a critique and reformulation. Am. Psychol. 42, 37–45 10.1037/0003-066X.42.1.37 3565913

[B50] Wei M. Heppner P. P. (2005). Counselor and client predictors of the initial working alliance. Couns. Psychol. 33, 51–71 10.1177/0011000004268636

[B51] Zhang N. Li Q. Yuan G. (2001). Expectation of folks on psychotherapy and counselling. Chin. Ment. Health J. 15, 250–252

